# Health Indicators as Measures of Individual Health Status, Their Perceived Importance, and Associated Factors: Cross-Sectional Observational Study

**DOI:** 10.2196/65616

**Published:** 2025-09-08

**Authors:** Temiloluwa Sokoya, Yuchun Zhou, Sebastian Diaz, Timothy D Law, Lina Himawan, Francisca Lekey, Lu Shi, Sarah F Griffin, Ronald W Gimbel, Xia Jing

**Affiliations:** 1 Department of Behavioral and Environmental Health, College of Health Sciences, Jackson State University, Jackson, MS, United States; 2 Gladys W and David H Patton College of Education, Ohio University, Athens, OH, United States; 3 College of Medicine, Northeast Ohio Medical University, Rootstown, OH, United States; 4 Ohio Musculoskeletal and Neurological Institute, Ohio University, Athens, OH, United States; 5 Department of Psychology, College of Arts and Sciences, Ohio University, Athens, OH, United States; 6 College of Health Sciences and Professions, Ohio University, Athens, OH, United States; 7 Department of Public Health Sciences, College of Behavioral, Social, and Health Sciences, Clemson University, Edwards Hall 519, PHS, CBSHS, Clemson, SC, 29634, United States, 1 8646563347

**Keywords:** health indicators, demographic factors, survey, public perspectives, correlation, multivariate analysis

## Abstract

**Background:**

Health indicators can facilitate the assessment of individual and population health status and are valuable in health care and public health settings. However, it is challenging to collect all measures at once. There is no single, objective, comprehensive, accurate, easy-to-use measure for individual health status yet, and we aim to develop one. We identified 29 health indicators via literature review and ranked their importance through public ratings to start the effort.

**Objective:**

This study aims to explore the effects of 4 demographic variables (age, sex, professional group, and educational level) on the importance ratings of the 29 indicators.

**Methods:**

This was a cross-sectional study. Online surveys were administered on 2 US college campuses and through ResearchMatch, an online platform specialized in finding clinical research participants. Only individuals 18 years or older were considered for inclusion as respondents. A 4-way multivariate analysis of variance (MANOVA) with post hoc testing was conducted to determine the effects of 4 demographic factors on the 29 health indicators. Descriptive statistics and bivariate correlation analysis were reported, along with the results of MANOVA with post hoc testing.

**Results:**

The study included 1153 participants from the United States of America with 1144 complete responses. Our study found significant correlations among the 29 health indicators. Age, education, sex, and professional groups showed correlations with multiple health indicators. Correlation analysis indicated that age was correlated with 16 of the 29 health indicators, followed by education (9 indicators), sex (8 indicators), and professional group (7 indicators). MANOVA modeling revealed that age (partial eta squared=0.058; *P*<.001), sex (partial eta squared=0.05; *P*<.001), professional group (partial eta squared=0.049; *P*<.001), and educational levels (partial eta squared=0.047; *P*<.001), as well as the interactions between sex and professional group (partial eta squared=0.044; *P*<.001) and sex and age (partial eta squared=0.041; *P*<.001), showed significant effects on the combination of the 29 health indicators. In general, female participants provided significantly higher ratings on 10 health indicators than male participants (*P*<.05), whereas older participants provided significantly higher ratings on 20 health indicators than younger participants (*P*<.05).

**Conclusions:**

Age emerged as a critical factor influencing the ratings of the 29 health indicators and showed the largest effect size along with sex, professional group, and educational level. These findings can be used as a quantitative foundation for the development of an objective measure for individual health status, for grouping health indicators, for conducting demographic factor adjustments during data analysis, for designing customized behavioral intervention studies based on demographic factors, or for health policy-making or program development.

## Introduction

### Contexts for Health Indicators

Health indicators can be used in various health care scenarios and public health settings, for example, measuring individual health status [1], measuring population health, community health, tracking longitudinal changes in individual or population health [2-4], tracking the outcomes of preventive services or programs over time, evaluating outcomes of interventions implemented, and comparing health statuses among more homogeneous subgroups [2,5-7]. The importance of health indicators has been recognized and studied since 1972 [8]. However, most of the effort has focused on group or population level measurements, for example, life expectancy and mortality [3,4,9,10]. Disease stages (eg, different stages of cancer) have long been used to help physicians determine more appropriate treatment plans and establish relatively more homogeneous patient groups for better comparability. For example, the Charlson Comorbidity Index (CCI), a comprehensive measurement that predicts long-term mortality, is one of the landmark tools used broadly to help stratify patients with chronic conditions into more homogeneous subgroups for better comparability and more accurate analysis [11,12].

### Limitations of the Current Health Indicators

The most used measurement of overall individual health status is self-rated health status, which has been shown to have good validity among the US population [5,13,14]. Although this measure had initially been used to predict mortality among older adults, published studies show its use in measuring outcomes of primary care [15], people with chronic conditions [16], and general adults [17]. Self-rated health status has also been broadly used by the World Health Organization (WHO) and the Organisation for Economic Co-operation and Development (OECD). Nevertheless, given the lack of clear criteria for rating each level of self-rated health status, the obtained ratings may be considered subjective. This subjectivity has been recognized as a potential limitation that could affect comparisons among different populations [5]. Numerous economists have focused on well-being studies and their measurements [18], using gross domestic product (GDP), GDP per capita, life expectancy, or mortality [19] as measures. Meanwhile, some researchers have focused on population health and socioeconomic determinants [18]. However, a literature search suggests the lack of a comprehensive, objective, and consistent measurement for healthy individuals or populations, that is, a counterpart of the CCI, albeit for healthy individuals instead of patients with chronic conditions, the primary target of the CCI.

### Needs of a Single, Objective, Comprehensive, and Easy-to-Use Health Indicator

Although life expectancy, morbidity, and mortality have long been used to measure and compare group health outcomes [20] within and among individual countries by the United Nations or WHO, more objective, comprehensive, and accurate methods for measuring a healthy individual’s health status are certainly needed, given the lack of existing measures. Some individual health indicators, such as BMI, blood pressure, drug and substance abuse, alcohol abuse, smoking, and tobacco use, may not be obtained easily all at once. Measurement of individual health status could provide important evidence to stratify the healthy population into more homogeneous subgroups, which can make comparisons meaningful.

### Our Previous Studies on Current Health Indicators

We started by conducting a literature review [1,5,21-25] to compile 29 health indicators [26] measuring an individual’s health status, including mental, physical, and social well-being. These health indicators include (in descending order based on public perceived importance rating): drug or substance abuse, smoking and tobacco use, alcohol abuse, major depression, diet and nutrition, blood sugar level, physical inactivity, immunization and vaccination, hypertension screening, low-density lipoprotein (LDL) cholesterol, blood triglycerides, high-density lipoprotein (HDL) cholesterol, having a sense of purpose in one’s life, cancer screening detection, total cholesterol, health literacy rate, personal care needs, air quality index > 100, family history of cancer, self-rated health status, HIV testing, insurance coverage, BMI, supply of dentists, sun protection, unemployed individual, engagement in life, high school diploma as a health indicator, and race and ethnicity [26].

**Figure 1.**
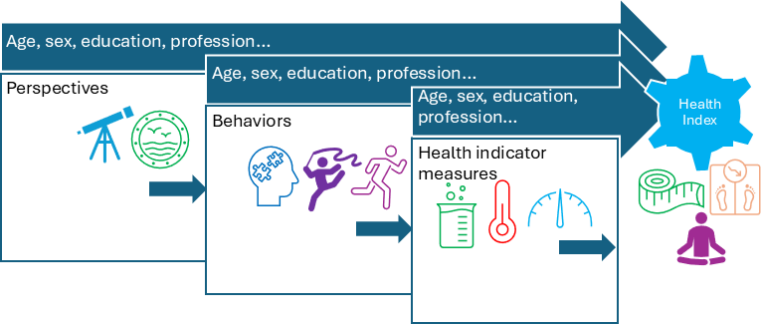
Conceptual framework between perspectives, health behaviors, health indicator measures, a comprehensive health index (our goal), and the contributing factors, eg, age, sex, education, profession, etc.

Thereafter, we examined 4 commercial electronic health record (EHR) systems, and the results showed that none of the systems included all 29 health indicators [27]. Practically, collecting all 29 health indicators can be burdensome for EHR users [28]. Then we conducted a survey wherein the public was invited to rate the importance of each health indicator. Such ratings may correspondingly impact their behaviors, though not necessarily consciously. Literature has documented people’s risk perception and its core impact on one’s health behavior [29,30]. The initial analysis of the importance ratings showed that the respondents agreed most on 13 of the 29 health indicators [26]. Figure 1 presents a conceptual framework illustrating the relationship between perspectives, health behaviors, health indicator measures, and associated factors, such as age, sex, education, and profession. This paper is a follow-up and in-depth analysis of the same survey results, focusing on the relationship between importance ratings and demographic information (respondents’ age, sex, education level, and professional group).

## Methods

### Study Design and Period

This was a cross-sectional study via 3 online surveys administered (Multimedia Appendix 1) at Ohio University (2017), ResearchMatch (2018), and Clemson University (2020). No compensation was provided to any respondents.

### Study Population

The only inclusion criterion in this study was that the survey respondents were required to be 18 years or older. The survey was administered via 2 public universities and ResearchMatch [31], which is an online platform for recruiting clinical research participants. Among the 1153 participants who responded, 1144 who provided complete responses for all question items were included as the final total sample.

### Data Collection Instrument

The survey was developed via different stages of testing and validation. The health indicators were grouped into 5 categories in the survey to provide a better experience for participants: health risks and behaviors (8 indicators), health care (3 indicators), health care provider supply (3 indicators), blood tests in physical exams (5 indicators), and others (10 indicators) [26]. The survey requested the respondents to rate the importance of 29 health indicators, with each health indicator being defined in the survey. The details related to the survey (see Multimedia Appendix 1) and definitions of each health indicator have all been published [26].

### Recruitment and Data Collection Procedures

Qualtrics surveys were used to administer the survey at Ohio University and Clemson University. The importance of each health indicator was rated from 1 (least important) to 10 (most important). The ResearchMatch survey was administered via REDCap (Research Electronic Data Capture; Vanderbilt University), with the ratings ranging from 1 (least important) to 100 (most important). The rating scale was converted into a 1 to 10 scale during the analysis.

The survey link was anonymous, and we explicitly stated in the invitations that the participants were welcome to share the link with others. Multimedia Appendix 2 shows the invitation email used in all 3 settings. All participants consented to the study electronically before they responded to the survey. At Ohio University and Clemson University, the survey was shared via an email broadcast. In ResearchMatch, the survey invitation was sent out randomly. All respondents were volunteers.

### Research Question for This Analysis

The following research question was used for this analysis: “What are the effects of age, sex, profession, and education on the perceived importance of 29 health indicators, individually or in combination, among the public?”

### Statistical Analysis Plan

This analysis examined all 29 health indicators according to age, sex, educational level, and professional group. Our analysis did not include race or ethnicity, mainly due to imbalanced data [26]. Descriptive statistics were reported to present the demographic profile of the participants (see Table 1).

**Table 1.** Descriptive statistics of all independent variable groups.

Independent variable		Results (N=1144), n (%)	
Sex			
Female		854 (74.65)	
Male		273 (23.86)	
Transgender		9 (0.79)	
Prefer not to answer		8 (0.7)	
Age group (years)			
≤35		460 (40.21)	
36-45		161 (14.07)	
46-55		151 (13.2)	
56-65		200 (17.48)	
>65		172 (15.04)	
Professional group			
Health care providers		227 (19.84)	
Public health professional		70 (6.12)	
Researcher who uses health indicator data		78 (6.82)	
Other researchers		203 (17.74)	
Other professional groups, specify		566 (49.48)	
Educational qualification			
High school		216 (18.88)	
Associate degree		95 (8.3)	
College degree		364 (31.82)	
Master’s degree		325 (28.41)	
Doctoral degree		144 (12.59)	

Specifically, to examine bivariate relationships among all variables, a correlation analysis was conducted among the 4 demographic factors and the 29 health indicators. To examine group mean differences, a complete multivariate analysis of variance (MANOVA) with the 4 demographic factors as the independent variables (IVs) and the 29 health indicators as the dependent variables (DVs) was performed. MANOVA was selected to test 29 health indicators simultaneously because it can test the combined effect of all IVs and all DVs at once rather than running separate tests. MANOVA helps control multiplicity by addressing the inflated type 1 error, and it is powerful for detecting multivariate patterns by considering the covariance structure of the 29 dependent variables.

Before we ran MANOVA, we tested the assumptions of normality and homogeneity of variances. Both assumptions were violated. The Box test results indicated significance. Levene test results for 13 of 29 dependent variables were significant. To address the violation of homogeneity, we used Pillai trace values and proceeded with Games-Howell post hoc testing. Regarding the violation of normality, we used MANOVA with no concerns because it is robust to violations of normality when the sample size is large (n=1144). For a sample size larger than 30, the central limit theorem says that the sample mean vectors are approximately multivariate normally distributed, even if the individual observations are not.

The null hypothesis of MANOVA was that a combination of the ratings for the 29 health indicators would have no significant mean difference between the groups. The alpha level for the 4-way MANOVA analysis was set as .05. As such, we identified significant 2-way interaction effects of age by sex and sex by professional group. Therefore, the final MANOVA model included 4 main effects of age, sex, education, professional group, and 2 interaction effects (ie, age by sex and sex by professional group). All statistical analyses were conducted via SPSS (version 29; IBM Corp).

### Ethical Considerations

This study was approved by the Ohio University Institutional Review Board (IRB; 17-X-142) and Clemson University IRB (IRB2019-441). All participants completed the consent form remotely before responding to the online survey. This study only uses aggregated results during analysis and in the communication of results; no identifiable information was used in any analysis or publication. The online survey is purely voluntary; no incentives were used except for a warm invitation email.

## Results

**Figure 2.**
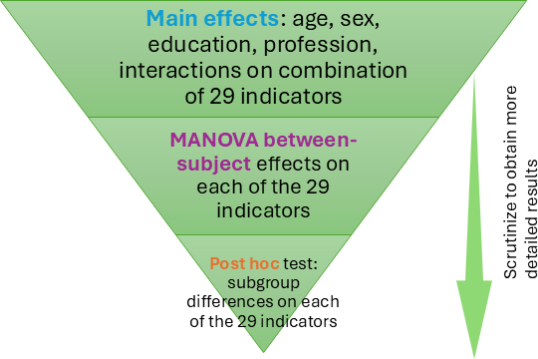
Organizational overview of the multivariate analysis results. MANOVA: multivariate analysis of variance.

Figure 2 organizes the results, excluding the participants’ profiles and the bivariate correlation results, to help readers navigate the Results section.

### Respondent Profile

Table 1 summarizes the percentages and numbers of all 4 independent variables (ie, age, sex, profession, and education) and their respective levels (ie, subgroups) to provide a basic profile of the survey respondents.

### Bivariate Correlations Between Demographic Factors and the 29 Health Indicators

The bivariate correlation analysis for age, sex, professional group, and education level, and the 29 health indicators (DV) indicated that all the dependent variables were significantly correlated with each other (Pearson correlation coefficient, *r*=0.058-0.838). The independent variable, age, was correlated with 21 of the dependent variables, except for the following 8 indicators: diet and nutrition, health literacy, high school diploma, HIV testing, immunization, insurance coverage, personal care needs, and total cholesterol level. Sex was correlated with 20 of the DVs, except for the following 9 indicators: air quality index >100, cancer screening, having a sense of purpose, immunization, high school diploma, insurance coverage, hypertension screening, personal care needs, and unemployment. Education level was correlated with these 11 health indicators, namely alcohol abuse, BMI, drug or substance abuse, physical inactivity, smoking, cancer screening, HIV testing, total cholesterol, engaged people, race and ethnicity, and unemployment. The professional group was correlated with 8 health indicators, namely diet and nutrition, engaged people, having a sense of purpose, high school diploma, personal care needs, physical inactivity, total cholesterol levels, and unemployment. The complete correlation matrix is provided in Multimedia Appendix 3.

### Main Effects of Demographic Factors on the Combination of the 29 Health Indicators

MANOVA modeling indicated that age, sex, professional group, and education level had significant main effects on the combination of 29 health indicators after controlling for other predictor effects. In addition, significant interaction effects between sex and professional group, as well as sex and age group, were observed on the 29 dependent variables after controlling for other predictor effects. The age group had a relatively increased effect size (ie, partial eta squared=0.058), whereas the interaction between sex and age group had a relatively small effect size (ie, partial eta squared=0.041).

Table 2 details the MANOVA results, including Pillai trace value, df, F, *P* values, and partial eta squared values. Only statistically significant interactions are presented in Table 2.

**Table 2.** Multivariate analysis of variance (MANOVA) results for the main effects of the 4 demographic factors on the combination of the 29 health indicators (only presents the combinations of demographic factors that are statistically significant, N=1114).

Effects	Pillai trace value	*F* test (*df*)	*P* value	Partial eta squared
Age group	0.232	2.304 (116)	<.001	0.058
Sex	0.149	1.951 (87)	<.001	0.050
Professional group	0.198	1.947 (116)	<.001	0.049
Educational qualification	0.189	1.859 (116)	<.001	0.047
Sex and professional group	0.352	1.731 (232)	<.001	0.044
Sex and age group	0.248	1.616 (174)	<.001	0.041

### Significant MANOVA Results for Between-Subject Effects

Four-way MANOVA with post hoc testing (Games-Howell) was conducted to examine group mean differences in the 29 health indicators according to the 4 demographic variables (age, sex, professional group, and education level). Table 3 presents the significant results of MANOVA modeling for between-subject effects. Accordingly, age showed significant effects on 16 health indicators, educational level on 9 health indicators, sex on 8 health indicators, and professional group on 7 health indicators. Meanwhile, the interactions between sex and age showed significant effects on 7 health indicators, whereas the interactions between sex and professional group showed significant effects on 5 health indicators.

**Table 3.** Significant multivariate analysis of variance (MANOVA) results for between-subject effects.

Independent and dependent variables		*F* test (*df*)	*P* value	Partial eta squared
Sex				
Alcohol abuse		4.304 (3)	.005	0.011
BMI		4.071 (3)	.007	0.011
Diet and nutrition		3.973 (3)	.008	0.011
Drug or substance abuse		3.597 (3)	.013	0.010
Physical inactivity		2.636 (3)	.048	0.007
Smoking or tobacco use		3.675 (3)	.012	0.010
Sun protection		2.642 (3)	.048	0.007
High school diploma as a health indicator		3.914 (3)	.009	0.010
Age group				
Alcohol abuse		2.912 (4)	.021	0.010
BMI		5.336 (4)	.000	0.019
Diet and nutrition		6.697 (4)	.000	0.024
Drug or substance abuse		4.189 (4)	.002	0.015
Family history of cancer		4.201 (4)	.002	0.015
Physical inactivity		4.103 (4)	.003	0.015
Smoking or tobacco use		2.444 (4)	.045	0.009
Sun protection		8.438 (4)	.000	0.029
Hypertension screening		4.240 (4)	.002	0.015
Self-rated health status		3.810 (4)	.004	0.014
Blood sugar level		2.914 (4)	.021	0.010
LDL^a^ cholesterol		2.663 (4)	.031	0.009
Air quality index >100		5.507 (4)	.000	0.019
Dentist supply		3.565 (4)	.007	0.013
Engaged people (people’s attention or efforts are occupied)		7.213 (4)	.000	0.025
Having a sense of purpose in one’s life		6.750 (4)	.000	0.024
Professional group				
Smoking or tobacco use		2.579 (4)	.036	0.009
Insurance coverage		3.284 (4)	.011	0.012
Personal care needs		3.616 (4)	.006	0.013
Self-rated health status		2.776 (4)	.026	0.010
Blood triglycerides		2.826 (4)	.024	0.010
Race and ethnicity		3.072 (4)	.016	0.011
Unemployed individual		2.981 (4)	.018	0.011
Educational qualification				
Alcohol abuse		2.511 (4)	.040	0.009
Family history of cancer		2.462 (4)	.044	0.009
Physical inactivity		2.831 (4)	.024	0.010
Smoking or tobacco use		3.041 (4)	.017	0.011
Cancer screening detection		3.413 (4)	.009	0.012
HIV testing		3.562 (4)	.007	0.013
Engaged people (people’s attention or efforts are occupied)		2.769 (4)	.026	0.010
Race and ethnicity		7.699 (4)	.000	0.027
Unemployed individual		4.897 (4)	.001	0.017
Sex and age group				
BMI		2.341 (6)	.030	0.012
Diet and nutrition		4.136 (6)	.000	0.022
Physical inactivity		3.502 (6)	.002	0.019
Smoking or tobacco use		2.195 (6)	.041	0.012
Air quality index >100		3.920 (6)	.001	0.021
Having a sense of purpose in one’s life		2.896 (6)	.008	0.015
Race and ethnicity		2.703 (6)	.013	0.014
Sex and professional group				
Smoking or tobacco use		2.149 (8)	.029	0.015
Insurance coverage		3.302 (8)	.001	0.023
Personal care needs		2.953 (8)	.003	0.021
Race and ethnicity		4.464 (8)	.000	0.031
Unemployed individual		2.026 (8)	.04	0.014

a LDL: low-density lipoprotein.

Table 3 details all significant MANOVA results for between-subject effects. The complete set of the MANOVA testing results of between-subject effects, including both significant and insignificant results, can be found in Multimedia Appendix 4.

### Post Hoc Testing Results

Of the 29 health indicators, 13 of them did not have homogenous variances. Due to this violation of the MANOVA assumption, we ran Games-Howell post hoc testing for each of them using ANOVA in SPSS. The other 16 indicators that met the equal variances assumption were tested using Bonferroni post hoc in SPSS. A close examination beyond the main effects of demographic factors on health indicator ratings through post hoc analysis revealed some interesting findings. More specifically, the MANOVA results for between-subject effects indicated significant differences in ratings between female and male respondents on the 10 health indicators (see Table 4, post hoc results and sex). All mean differences between these 10 health indicators showed that female ratings were higher than male ratings. The full set of post hoc testing results is available upon request.

**Table 4.** The significant multivariate analysis of variance (MANOVA) results and post hoc testing results.

	Main effects and interaction effects						Post hoc results			
Health indicators	Sex	Age	Professional	Education	Sex by age	Sex by professional	Sex^a^	Age^b^	Profession^c^	Education^d^
Games-Howell post hoc testing results for the 13 health indicators with heterogeneous variances										
HIV testing^e^	—^f^	—^g^	—	✓^h^	—	—	1>2^h^	—	—	1>4, 1>5^h^
Blood sugar^e^ level	—^f^	✓^h^^,^^f^	—	—	—	—	1>2^h^	3>1, 4>1, 5>1, 4>2, 5>2^h^	—	—
Alcohol abuse	✓^h^	✓^h^	—	✓^h^	—	—	1>2^h^	3>1, 4>1, 5>1, 5>2^h^	—	3>1, 5>1^h^
BMI	✓^h^	✓^h^	—	—	✓^h^	—	—	3>1, 4>1, 5>1, 4>2, 5>2^h^	—	—
Diet and nutrition	✓^h^	✓^h^	—	—	✓^h^	—	1>2^h^	—	—	—
Drug and substance abuse	✓^h^	✓^h^	—	—	—	—	1>2^h^	3>1, 4>1, 5>1, 4>2, 5>2^h^	—	5>1^h^
Physical inactivity	✓^h^	✓^h^	—	✓^h^	✓^h^	—	—	2>1, 4>1^h^	1>5^h^	3>1, 4>1, 5>1^h^
Smoking	✓^h^	✓^h^	✓^h^	✓^h^	✓^h^	✓^h^	1>2^h^	2>1, 3>1, 4>1, 5>1^h^	3>4, 3>5^h^	3>1, 4>1, 5>1^h^
Insurance coverage	—	—	✓^h^	—	—	✓^h^	1>2^h^	—	2>1, 2>5^h^	—
High School	✓^h^	—	—	—	—	—	—	—	—	—
Engaged people	—	✓^h^	—	✓^h^	—	—	—	3>1, 4>1, 5>1, 4>2, 5>2, 5>3^h^	1>3^h^	3>1, 4>1, 5>1^h^
Major depression	—	—	—	—	—	—	—	4>1^h^	—	—
Have a sense of purpose in life	—	✓^h^	—	—	✓^h^	—	—	4>1, 5>1, 4>2, 5>2^h^	1>4, 1>5^h^	—
Bonferroni post hoc testing results for the 16 health indicators with homogenous variances										
Immunization^e^	—	—	—	—	—	—	—	—	—	—
Personal care needs^e^	—	—	✓^h^	—	—	✓^h^	—	—	—	—
Cancer screening^e^	—	—	—	✓^h^^,^^f^	—	—	—	—	—	—
Self-rated health status^e^	—	✓^h^^,^^f^	✓^h^	—	—	—	—	5>1, 5>2^h^	—	—
Air quality index^e^	—	✓^h^^,^^f^	—	—	✓^h^	—	—	4>1, 5>1, 4>2^h^	—	—
Dentist supply^e^	—^f^	✓^h^^,^^f^	—	—	—	—	1>2^h^	4>1, 5>1, 4>2^h^	—	—
Health literacy rate^e^	—^f^	—	—	—	—	—	—	—	—	—
Blood triglycerides^e^	—^f^	—^f^	✓^h^	—	—	—	—	5>1, 5>2^h^	—	—
HDL^i^ cholesterol^e^	—^f^	—	—	—	—	—	—	5>1^h^	—	—
LDL^j^ cholesterol^e^	—^f^	✓^h^^,^^f^	—	—	—	—	—	5>1, 5>2^h^	—	—
Total cholesterol^e^	—	—	—	—^f^	—	—	—	—	—	—
Family history of cancer	—	✓^h^	—	✓^h^	—	—	—	3>1, 4>1, 5>1, 4>2^h^	—	—
Sun protection	✓^h^	✓^h^	—	—	—	—	1>2^h^	3>1, 4>1, 5>1, 4>2, 5>2^h^	5>4^h^	—
Hypertension screening	—	✓^h^	—	—	—	—	—	3>1, 4>1, 5>1, 4>2, 5>2^h^	—	—
Race and ethnicity	—	—	✓^h^	✓^h^	✓^h^	✓^h^	1>2^h^	4>1, 5>1^h^	2>4, 2>5, 3>4, 3>5^h^	2>1, 3>1, 4>1, 5>1^h^
Unemployed individual	—	—	✓^h^	✓^h^	—	✓^h^	—	5>1^h^	—	3>1, 4>1, 5>1^h^

a 1: Female; 2: Male; 1>2 indicates female rated higher.

b 1:≤35 years; 2: 36‐45 years; 3: 46‐55 years; 4: 56‐65 years; 5:>65 years.

c 1: Healthcare providers; 2: Public health professional; 3: Researcher who uses health indicator data; 4: Other researchers; and 5: Other professional groups, specify.

d 1: High school; 2: Associate degree; 3: College degree; 4: Master's degree; 5: Doctoral degree.

e The 13 health indicators with more consistent ratings.

f *P*<.05 when examining the 13 health indicators with more consistent ratings.

g Nonsignificant result.

h *P*<.05 when examining 29 indicators.

i HDL: high-density lipoprotein.

j LDL: low-density lipoprotein.

Regarding age, post hoc testing results showed that the ratings for the following 20 health indicators all showed a similar trend of significantly higher ratings among older participants than among younger participants: self-rated health status, air quality index >100, dentist supply, blood sugar level, blood triglycerides, HDL, LDL, alcohol abuse, BMI, drug or substance abuse, family history of cancer, physical inactivity, smoking, sun protection, hypertension screening, engaged people, major depression, having a sense of purpose, race and ethnicity, and unemployment (see Table 4, post hoc results and age).

Post hoc testing results also showed that health care providers rated 3 health indicators significantly higher than other professional groups: physical inactivity, engaged people, and having a sense of purpose in life (see Table 4, post hoc results, and profession). Meanwhile, public health professionals rated 2 health indicators significantly higher than other professional groups: insurance coverage and race and ethnicity. More complete results are presented in Table 4.

Post hoc testing results showed that participants with higher educational levels provided significantly and consistently higher ratings for the following 7 health indicators than those with lower educational levels: alcohol abuse, drug and substance abuse, physical inactivity, smoking, engaged people, race and ethnicity, and unemployment (see Table 4, post hoc results and education). Meanwhile, participants with high school education rated significantly higher for HIV testing than those with Master’s or Doctoral degrees.

### Overlapping Results According to Different Approaches

The left part of Table 4 (see Main effects and interaction effects) presents an alternative view of a portion of the results in Table 3. The left side of Table 4 also combines the results when the same approach was used, but only examined the 13 more consistent health indicators per our previous publication [26] (Table 4). After considering the results from all 29 health indicators and the 13 more consistently rated health indicators, we found that age apparently showed consistent and significant effects on the ratings of the following 15 health indicators: blood sugar level, alcohol abuse, BMI, drug and substance abuse, physical inactivity, smoking, engaged people, having a sense of purpose in life, self-rated health status, air quality index >100, dentist supply, LDL, family history of cancer, sun protection, and hypertension screening. In other words, older participants provided higher ratings for these indicators than did younger participants. Meanwhile, the educational level also showed a significant effect on the ratings of HIV testing (inversely), alcohol abuse, physical inactivity, smoking, engaged people, race and ethnicity, and unemployment. Except for HIV testing, all 6 other health indicators were observed such that participants with higher educational levels provided higher ratings for them.

In addition, we also noticed the following interesting findings from Table 4:

1. After examining all 29 health indicators, we found that 5 indicators did not show any significant effects according to demographic factors or their interactions: immunization, health literacy rate, HDL, total cholesterol, and major depression (see Table 4).
2. Meanwhile, there are 3 health indicators showing the significant effects of 4 or 5 demographic factors and their interactions: smoking (5), physical inactivity (4), and race and ethnicity (4).
3. Among the 29 indicators, 13 that received more consistent ratings showed much fewer significant effects according to demographic factors and their interactions:
  - A total of 12 significant effects for 13 indicators versus 40 significant effects for the rest of the 16 indicators, that is, the “Main effects and interaction effects" in Table 4.
  - A total of 11 significant post hoc results for 13 indicators versus 34 significant post hoc results for the rest of 16 indicators, that is, the "Post hoc results" in Table 4.

## Discussion

### Interpretation of Results

In summary, female participants rated 10 health indicators higher than male participants, and older participants rated 20 health indicators higher than younger participants, based on an overlap analysis of different statistical methods. The demographic factors showed the least significant effects on these 5 health indicators: immunization, health literacy rate, HDL, total cholesterol, and major depression. Meanwhile, demographic factors show significant impacts on ratings of smoking, physical activity, and race and ethnicity.

An alternative presentation of the MANOVA analysis is provided in Table 4 (ie, the "Main effects and interaction effects" from Table 3, whereas the "Post hoc results" details the significant results from post hoc testing); we also noticed the following points. First, the main effects may not be significant when significant post hoc test results were obtained, possibly due to cancellation effects. Second, post hoc results are always necessary when using a complicated MANOVA model for comparisons among subgroups, which cannot be demonstrated via the main effects. Third, among the 29 health indicators, 13 that showed more consistent importance ratings had much fewer main effects and fewer significant post hoc test results than did the remaining 16 health indicators. This third point validates the results across different analytic strategies.

### Significance of the Work

This study has endeavored to prioritize the 29 commonly used health indicators via public perspective surveys, thereby providing a foundation for the prioritization of these 29 health indicators, 13 of which received relatively consistent ratings across all 3 samples. The detailed results can be used to determine which health indicators should be included in EHR or PHR systems, given the burden associated with collecting all 29 health indicators. The survey results [26] and further analysis presented in this study provide a foundation for developing a comprehensive health indicator formula that can measure individual health and outcomes of preventive medicine services over time [21,32-34]. Preventive services have been recognized as important for controlling the increasing costs of health care [35,36]. However, the accurate measurement of such services can be challenging given the complexity of measuring most of their outcomes within a short time period.

This paper further explored demographic factors, namely age, sex, educational level, and professional group, and their effects on public ratings of these 29 health indicators. Our results showed that age was a critical factor, with older participants rating 20 health indicators significantly higher than younger ones, indicating the wisdom accumulated based on experience and time. However, more carefully designed experiments are required to determine the actual relationship between age and importance ratings of the 20 health indicators, which is beyond the scope of this paper. However, our results can provide quantitative evidence for age-based adjustments during health indicator-related data analysis.

Sex also played a critical role in ratings of 10 health indicators, with female participants providing significantly higher ratings than male participants. Considering the longer life expectancy among females than males in many countries (5.4 y difference in the United States) [37] and the relationship between perception and health behavior, our public perspective results in the importance ratings of the 10 health indicators aligning well with the life expectancy difference between females and males. Our understanding is that females may pay more attention to their health in general, as evidenced by more female participants in our study and in other studies, higher ratings of health indicators, more female participants in fitness group exercises, and longer life expectancies. However, the actual and accurate relationship may not be as linear, given that females perceived these health indicators to be more important than males, which could lead to healthier behaviors and, eventually, a longer life expectancy. Nonetheless, the observed data points seemingly demonstrate a trend. In addition, we also believe that this point validates our public perspective survey results. Regardless of whether such gender-related differences have physiological roots or purely sociocultural effects [38,39], similar observations were documented between females and males. Our results could be used in making health-related policy, especially in the public health field, for example, conducting more intensive campaigns focusing on males or promoting healthy lifestyles among males. Perhaps new ways of delivering health-related information need to be explored to better engage males during their lifespan.

The detailed comparisons and results presented in this paper can be used as a quantitative foundation to develop a more objective and comprehensive measure of individual health status. In addition, our results can be used as a foundation for designing future health behavioral studies, considering the published demonstration of relationships between perceptions and health behavior [29,30]. The public’s perspectives on the studied health indicators have been assumed to be influenced by their health-related awareness or behaviors, either consciously or subconsciously. Our study results could shed some light on the adjustment of demographic-related factors during data analysis or modeling of health-related factors.

### Comparison With Other Similar Measures to Provide Context for Our Results

County Health Ranking & Roadmaps, a typical population-level program established by the University of Wisconsin Population Health Institute and supported by the Robert Wood Johnson Foundation [40], compares 2 categories (health outcomes and health factors), 5 classes (health outcomes, health behaviors, clinical care, social & economic factors, and physical environment), and 15 measures and demographic information for each county in the United States [40]. Several similarities in the measures used have been noted between our study (to measure individual health status) and the mentioned program. In fact, a comparison showed a 60% (9/15) overlap in the measures used, which provides a sense of validation and methodological alignment for our study, considering County Health Ranking & Roadmaps is an established and well-respected project, and their results were used and cited broadly in developing population-level health programs and policies. Meanwhile, we also noticed that the main differences were that County Health Rankings used more measures in the social and economic factors, such as income, housing, family and social support, and community safety, whereas our study used more individual-level measures, such as blood sugar level and total cholesterol. Considering that focused groups vary quite significantly (ie, individuals versus county-level populations), the difference in measurement selection is understandable despite similarly attempting to measure health. Our study and results provide a complementary role to the County Health Ranking & Roadmaps, which focuses on the health and comparison between counties, a basic unit, while our study focuses on individuals’ health. Considering our different application purposes, we think our study and County Ranking & Roadmaps can be used in complementary ways to study individual or their collective health status.

The UK Health Index, which consists of 3 categories, namely healthy people, healthy lives, and healthy places, has been used to track changes over time and compare the health of residents across different areas of England [2,6,41]. The healthy lives category is very similar to many of our health indicators, also referred to as modifiable risk factors, at the individual level. The healthy people category measures health at the population level, whereas the healthy places category measures health at the system level or beyond individual control (eg, air quality) [42].

A closer comparison of the UK Health Index [6,41] and our 29 health indicators [26] showed a large overlap in 15 health indicators between the two. Over half of the health indicators used in our study were also used by the UK Health Index, which provides additional validity for these health indicators, considering that the UK Health Index is a government-funded project.

However, after closely examining the methodology used in developing the UK Health Index, we noticed the following differences between our project and the UK Health Index project. First, the UK Health Index was developed to measure a nation’s health status at both the population and individual levels, whereas our health indicators were used to measure individual health status only. Collectively, however, such indicators can be aggregated to compare health outcomes across different groups or track changes over time. Second, in terms of the long-term objectives, our goal was to use the survey results, analysis results, and real-world data to develop a formula that could calculate a more objective, data-driven health indicator to measure an individual’s overall health status. The UK Health Index project used factor analysis to group health indicators and determine the weight of each health indicator. Third, as a next-stage effort, our project will focus on formula development and validation of health indicators, whereas the UK Health Index project uses existing and available data sources and already provides Health Index comparisons among various areas in England, as well as comparisons for the same area over time. Although the references provided by the UK Health Index project are exceedingly helpful [42,43], our study’s focus and trajectory differ from that of the UK Health Index project. Fourth, given our initial goal of measuring individual health status, measurement is our core focus, which will be necessary for the long-term use of our study within EHR or PHR systems. The UK Health Index, however, focuses on the availability and continuity of data sources and compares the same or various locations across different time points.

### Strengths and Weaknesses of the Study

Our study explores strategies for measuring individual health more objectively, accurately, and comprehensively. Currently, an objective and accurate measure of individual health status is missing. Such a measure can track the performance of health care systems, preventive services, or intervention programs for individuals, as well as the health and outcomes of the population over time. In addition, such measures can be used to establish more comparable and homogeneous subgroups for conducting epidemiological or health service studies. Although our study constitutes the first step in the development of a larger project, its direction is critical for improving the health of the whole population and the use and promotion of preventive care services. In addition, we used multiple statistical methods during the results analysis, and we emphasized the overlap results across different methods, which provides additional confidence in the results. We feel this is an important step to mitigate the potential limitations of observational studies like ours.

Our work is in the preliminary stages, which is the main limitation of our study. Although our survey included a validation question and only included complete and valid responses in the analysis, this analysis was based on public perspectives. Despite this reality, we believe that our deep analysis of the demographic variables of individual respondents and their effects on and interactions with the rating results are nevertheless helpful for thoroughly understanding the public’s perspectives on the 29 health indicators. In addition, our analysis and results can provide insights and quantitative evidence for the future development of a comprehensive individual health index.

Another limitation was that our results were derived from survey results. Despite the relatively large sample sizes and in-depth analysis, our study is an observational study. Nonetheless, our results can shed light on the correlations between various demographic subgroups and ratings. However, given that they are not as convincing as those from a hypothesis-driven randomized controlled study, we urge readers to interpret the results within context. In addition, other factors, such as pain or sleep, had not been considered in our study [10]. Meanwhile, we acknowledge that, due to the voluntary nature of the survey, the respondents have a higher percentage of female participants with relatively higher educational levels compared to the general public. Therefore, users need to put the results into these contexts. In addition, the use of different scales (1‐10 in Qualtrics and 1‐100 in REDCap) may also lead to unintended effects in ratings.

### Potential Future Developments

Our next step is to explore longitudinal individual health data, possibly through EHR, to establish comparable cohorts to develop, refine, and validate the new individual health measure. All of Us, a National Institutes of Health—initiated project, can be a potential platform and data source to develop and validate the measure [44,45]. Despite numerous studies exploring a measure of health status for decades [8,9,46-49], no consensus has yet been reached regarding such a measure. In addition, others can use our results during data analysis, especially when they need to conduct age- or sex-based adjustments. Meanwhile, our results can also be used as evidence to develop more customized public health programs or interventional studies related to the demographic factors we analyzed in this study. In addition, designing further studies to understand how demographic factors affect the importance ratings of health indicators is certainly worthwhile to explore.

### Conclusion

Our analysis showed that all 29 health indicators were significantly intercorrelated. The MANOVA modeling results indicated that age, sex, professional group, educational level, and the interaction between sex and professional group and sex and age had significant main effects on the combination of 29 health indicators. Our findings could provide a quantitative foundation for (1) developing a formula for an overall health index, (2) providing evidence to guide the design of customized behavioral interventional studies for subgroups of the public, and (3) providing evidence for demographic factor-related adjustments during data analysis, modeling, policy making, and program development.

## Supplementary material

10.2196/65616 Multimedia Appendix 1 A copy of the online survey used to obtain public perspectives on health indicators.

10.2196/65616 Multimedia Appendix 2 The invitation message used to invite participants to complete the online survey.

10.2196/65616 Multimedia Appendix 3 The correlation matrix among the 4 demographic factors and 29 health indicators.

10.2196/65616 Multimedia Appendix 4 The full version of Table 3 (statistically significant and insignificant results). Multivariate analysis of variance (MANOVA) results for the main effects and interaction effect of demographic factors on the 29 individual health indicators.

## Abbreviations

CCI: Charlson Comorbidity Index
DV: dependent variable
EHR: electronic health record
GDP: gross domestic product
HDL: high-density lipoprotein
IV: independent variable
LDL: low-density lipoprotein
MANOVA: multivariate analysis of variance
OECD: Organization for Economic Co-operation and Development
WHO: World Health Organization
