# Supplementary material for: Health Indicators as Measures of Individual Health Status, Their Perceived Importance, and Associated Factors: Cross-Sectional Observational Study
Source: JMIR Public Health Surveill. 2025 Sep 8;11:e65616. doi: 10.2196/65616 (PMC12439059; doi:10.2196/65616)
Supplement: Multimedia Appendix 2 [file publichealth-v11-e65616-s002.pdf]

Dear friend:

Good day!

If you are 18 years or older, you are cordially invited to share your perspectives about what can be used to measure an individual's health status (as opposed to disease status), i.e., **health indicators,** and their importance. Individual **health status** refers to a person's overall physical, mental, and social well-being, free from illness or injury. Individual **disease status** refers to a person who has physical or mental symptoms with or without definite diagnoses. Your perspective will help to provide Clemson University researchers' with a foundation for the more consistent, accurate, and convenient measurement of preventive services. It should take 10 - 15 minutes to complete the survey. You are more than welcome to ***share the link*** with your friends and family.

We look forward to your input! The survey link is:

https://clemson.ca1.qualtrics.com/jfe/form/SV_9LjjhOHqm3j7bDv

The study has been exempted by Clemson University IRB (IRB2019-441).

For any questions, suggestions or comments about the survey or the project, please contact *Xia Jing,* xjing@clemson.edu *or 864-656-3347, or Ron Gimbel (Professor of the Department of Public Health Sciences),* rgimbel@clemson.edu*, or by 863-656-1969, or Lu Shi (Associate Professor of the Department of Public Health Sciences),* lus@clemson.edu*, or by 864-656-0495.*

This survey is being distributed with the approval of the Office for Institutional Assessment at Clemson University. For more information regarding surveys at Clemson University, please visit their website at https://www.clemson.edu/assessment/surveys/. To view the Data Management Plan for this survey, please visit the link below:

https://clemson.box.com/s/wkbj0gruqkr9luqwqj4owz0o35loxt7j

Thank you in advance for your time and participation!

Sincerely,

Xia, Ron, and Lu

==

Xia Jing CLEMSON UNIVERSITY
Assistant Professor
Department of Public Health Sciences
864.656.3347
511 Edwards Hall

College of Behavioral, Social and Health Sciences
Building People and Communities
