# Supplementary material for: Health Indicators as Measures of Individual Health Status, Their Perceived Importance, and Associated Factors: Cross-Sectional Observational Study
Source: JMIR Public Health Surveill. 2025 Sep 8;11:e65616. doi: 10.2196/65616 (PMC12439059; doi:10.2196/65616)
Supplement: Multimedia Appendix 3 [file publichealth-v11-e65616-s003.pdf]

Correlation matrix of all variables in the study

		Age Group	Gender	professional group	educational qualification	Race	Alcohol abuse	Body mass index (BMI)	Diet and nutrition	Drug or substance abuse	Family history of cancer	Physical inactivity	Smoking, tobacco use	Sun protection	Immunization/Vaccination	Insurance coverage	Personal care needs	Cancer screening detection	Hypertension screening	HIV testing	Self-rated health status	Blood sugar level	Blood triglycerides	HDL cholesterol	LDL cholesterol	Total cholesterol	High school diploma as a health indicator	Air quality index > 100	Dentist supply	Engaged people (people's attention or efforts are occupied)	Health literacy rate	Major depression	Having a sense of purpose in one's life	Race and ethnicity	Unemployed individual
Age Group	Pearson Correlation	--																																	
Gender	Pearson Correlation	0.004	--																																
professional group	Pearson Correlation	.065*	.069*	--																															
educational qualification	Pearson Correlation	.268**	-0.014	-.111**	--																														
Race	Pearson Correlation	-.117**	.135**	0.005	.064*	--																													
Alcohol abuse	Pearson Correlation	.175**	-.112**	0.023	.085**	-0.001	--																												
Body mass index (BMI)	Pearson Correlation	.233**	-.113**	-0.035	.070*	0.007	.216**	--																											
Diet and nutrition	Pearson Correlation	-0.038	-.134**	-.058*	0.033	-0.027	.288**	.318**	--																										
Drug or substance abuse	Pearson Correlation	.191**	-.142**	-0.001	.081**	0.009	.707**	.250**	.285**	--																									
Family history of cancer	Pearson Correlation	.185**	-.099**	0.056	-0.030	0.013	.261**	.256**	.230**	.303**	--																								
Physical inactivity	Pearson Correlation	.088**	-.063*	-.096**	.150**	-.060*	.265**	.265**	.529**	.274**	.310**	--																							
Smoking, tobacco use	Pearson Correlation	.191**	-.137**	-0.051	.154**	-0.031	.511**	.252**	.357**	.589**	.326**	.457**	--																						
Sun protection	Pearson Correlation	.249**	-.165**	0.021	0.030	-.068*	.267**	.264**	.300**	.239**	.478**	.379**	.362**	--																					
Immunization/Vaccination	Pearson Correlation	0.023	-0.001	0.045	-0.027	0.021	.114**	.067*	.101**	.146**	.286**	.118**	.156**	.323**	--																				

Insurance coverage	Pearson Correlation	0.007	-0.057	0.011	0.056	0.007	0.038	.062*	.085**	0.036	.111**	.136**	.070*	.217**	.296**	--																			
Personal care needs	Pearson Correlation	0.014	-0.035	-.062*	-0.039	0.030	.122**	.118**	.224**	.127**	.206**	.251**	.108**	.317**	.290**	.465**	--																		
Cancer screening detection	Pearson Correlation	.082**	-0.044	0.026	-.095**	0.000	.204**	.195**	.217**	.193**	.469**	.215**	.204**	.448**	.425**	.263**	.331**	--																	
Hypertension screening	Pearson Correlation	.197**	-0.054	-0.014	0.037	-0.020	.208**	.232**	.211**	.213**	.391**	.238**	.257**	.418**	.381**	.258**	.303**	.744**	--																
HIV testing	Pearson Correlation	-0.044	-.103**	0.031	-.135**	0.049	.204**	.146**	.219**	.203**	.399**	.217**	.212**	.408**	.318**	.237**	.333**	.602**	.551**	--															
Self-rated health status	Pearson Correlation	.126**	-.059*	-0.019	-0.023	0.008	.149**	.101**	.144**	.139**	.127**	.160**	.114**	.222**	.126**	.194**	.300**	.185**	.169**	.175**	--														
Blood sugar level	Pearson Correlation	.182**	-.078**	-0.023	0.057	0.046	.286**	.266**	.304**	.302**	.363**	.322**	.310**	.346**	.282**	.180**	.301**	.426**	.499**	.366**	.223**	--													
Blood triglycerides	Pearson Correlation	.124**	-.060*	0.019	0.009	0.040	.266**	.290**	.286**	.252**	.390**	.286**	.249**	.372**	.313**	.150**	.305**	.444**	.492**	.402**	.221**	.733**	--												
HDL cholesterol	Pearson Correlation	.082**	-.090**	0.033	-0.012	0.017	.260**	.246**	.269**	.260**	.376**	.296**	.264**	.387**	.313**	.139**	.316**	.453**	.486**	.422**	.172**	.663**	.822**	--											
LDL cholesterol	Pearson Correlation	.118**	-.105**	0.014	0.043	0.020	.265**	.292**	.261**	.250**	.368**	.311**	.303**	.374**	.319**	.164**	.311**	.463**	.503**	.404**	.203**	.677**	.797**	.838**	--										
Total cholesterol	Pearson Correlation	0.051	-.090**	.059*	-.060*	0.034	.245**	.279**	.255**	.215**	.359**	.270**	.230**	.363**	.326**	.145**	.282**	.444**	.445**	.393**	.209**	.621**	.715**	.740**	.779**	--									
High school diploma as a health indicator	Pearson Correlation	-0.017	-0.025	-.068*	0.029	-.070*	0.019	.058*	.114**	0.017	.092**	.135**	0.043	.143**	.167**	.430**	.257**	.152**	.126**	.118**	.250**	.098**	.117**	.109**	.138**	.112**	--								
Air quality index > 100	Pearson Correlation	.120**	-0.009	0.037	-0.016	.064*	.171**	.155**	.289**	.154**	.249**	.257**	.188**	.340**	.230**	.253**	.345**	.321**	.299**	.295**	.276**	.292**	.301**	.292**	.294**	.239**	.268**	--							
Dentist supply	Pearson Correlation	.169**	-.117**	0.052	-0.010	-0.024	.187**	.140**	.218**	.155**	.264**	.219**	.184**	.413**	.314**	.324**	.371**	.408**	.379**	.360**	.336**	.358**	.358**	.344**	.349**	.334**	.336**	.572**	--						
Engaged people (people's attention or efforts are occupied)	Pearson Correlation	.248**	-.103**	-.061*	.131**	-0.039	.181**	.199**	.222**	.162**	.167**	.224**	.183**	.298**	.148**	.239**	.311**	.243**	.260**	.164**	.317**	.226**	.232**	.225**	.232**	.168**	.304**	.401**	.471**	--					

Health literacy rate	Pearson Correlation	0.031	-.066*	-0.048	0.042	-0.010	.129**	.149**	.266**	.106**	.136**	.260**	.134**	.271**	.227**	.314**	.343**	.276**	.286**	.231**	.268**	.275**	.270**	.260**	.239**	.217**	.377**	.436**	.457**	.514**	--				
Major depression	Pearson Correlation	.092**	-.060*	-0.026	0.003	-0.022	.377**	.147**	.278**	.352**	.236**	.283**	.297**	.304**	.218**	.159**	.282**	.303**	.335**	.352**	.238**	.324**	.296**	.317**	.293**	.298**	.163**	.293**	.313**	.358**	.332**	--			
Having a sense of purpose in one's life	Pearson Correlation	.167**	-0.043	-.081**	0.045	0.003	.214**	.160**	.273**	.194**	.142**	.266**	.204**	.250**	.113**	.178**	.291**	.197**	.229**	.184**	.333**	.198**	.199**	.223**	.218**	.188**	.297**	.345**	.315**	.539**	.363**	.460**	--		
Race and ethnicity	Pearson Correlation	.142**	-.096**	-0.058	.167**	-0.028	.071*	.139**	.126**	0.031	.226**	.130**	.101**	.292**	.185**	.348**	.219**	.243**	.269**	.193**	.188**	.191**	.190**	.196**	.217**	.181**	.350**	.253**	.279**	.304**	.296**	.180**	.241**	--	
Unemployed individual	Pearson Correlation	.098**	-0.047	-.071*	.147**	-0.002	.134**	.115**	.162**	.105**	.103**	.178**	.142**	.241**	.125**	.402**	.283**	.190**	.242**	.200**	.238**	.197**	.198**	.204**	.197**	.176**	.423**	.313**	.356**	.423**	.393**	.341**	.402**	.512**	--

*. Correlation is significant at the 0.05 level (2-tailed).
**. Correlation is significant at the 0.01 level (2-tailed).
