# Supplementary material for: Health Indicators as Measures of Individual Health Status, Their Perceived Importance, and Associated Factors: Cross-Sectional Observational Study
Source: JMIR Public Health Surveill. 2025 Sep 8;11:e65616. doi: 10.2196/65616 (PMC12439059; doi:10.2196/65616)
Supplement: Multimedia Appendix 4 [file publichealth-v11-e65616-s004.pdf]

Tests of Between-Subjects Effects

Source	Dependent Variable	Type III Sum of Squares	df	Mean Square	F	Sig.	Partial Eta Squared
Corrected Model	Immunization/Vaccination	172.014[a]	31	5.549	1.147	0.266	0.031
	Personal care needs	243.593[b]	31	7.858	1.733	0.008	0.046
	Cancer screening detection	181.855[c]	31	5.866	1.281	0.140	0.034
	HIV testing	420.707[d]	31	13.571	2.324	0.000	0.061
	Self-rated health status	296.092[e]	31	9.551	2.100	0.000	0.055
	Air quality index > 100	238.644[f]	31	7.698	2.000	0.001	0.053
	Dentist supply	368.659[g]	31	11.892	2.831	0.000	0.073
	Health literacy rate	219.295[h]	31	7.074	1.633	0.016	0.044
	Blood sugar level	194.862[i]	31	6.286	2.172	0.000	0.057
	Blood triglycerides	163.004[j]	31	5.258	1.495	0.040	0.040
	HDL cholesterol	192.243[k]	31	6.201	1.695	0.010	0.045
	LDL cholesterol	245.996[l]	31	7.935	2.163	0.000	0.057
	Total cholesterol	206.455[m]	31	6.660	1.658	0.014	0.044
	Alcohol abuse	293.459[n]	31	9.466	2.946	0.000	0.076
	Body mass index (BMI)	681.961[o]	31	21.999	4.063	0.000	0.102
	Diet and nutrition	234.688[p]	31	7.571	2.726	0.000	0.071
	Drug or substance abuse	270.258[q]	31	8.718	3.137	0.000	0.080
	Family history of cancer	404.995[r]	31	13.064	3.049	0.000	0.078
	Physical inactivity	302.508[s]	31	9.758	3.016	0.000	0.078
	Smoking, tobacco use	403.678[t]	31	13.022	4.577	0.000	0.113
	Sun protection	691.143[u]	31	22.295	5.332	0.000	0.129
	Insurance coverage	511.020[v]	31	16.485	2.058	0.001	0.054
	Hypertension screening	310.492[w]	31	10.016	2.456	0.000	0.064
	High school diploma as a health indicator	344.698[x]	31	11.119	1.444	0.056	0.039
	Engaged people (people's attention or efforts are occupied)	803.651[y]	31	25.924	4.397	0.000	0.109
	Major depression	166.185[z]	31	5.361	1.863	0.003	0.049
	Having a sense of purpose in one's life	412.749[aa]	31	13.314	2.926	0.000	0.075
	Race and ethnicity	928.797[ab]	31	29.961	4.612	0.000	0.114
	Unemployed individual	493.162[ac]	31	15.908	2.676	0.000	0.069
Intercept	Immunization/Vaccination	1983.994	1	1983.994	410.279	0.000	0.270
	Personal care needs	1391.102	1	1391.102	306.764	0.000	0.216
	Cancer screening detection	1928.917	1	1928.917	421.161	0.000	0.275
	HIV testing	1602.802	1	1602.802	274.437	0.000	0.198
	Self-rated health status	1461.112	1	1461.112	321.215	0.000	0.224
	Air quality index > 100	1526.277	1	1526.277	396.444	0.000	0.263
	Dentist supply	1286.673	1	1286.673	306.285	0.000	0.216
	Health literacy rate	1524.749	1	1524.749	352.000	0.000	0.240
	Blood sugar level	2117.562	1	2117.562	731.606	0.000	0.397
	Blood triglycerides	2007.957	1	2007.957	571.000	0.000	0.339
	HDL cholesterol	1840.828	1	1840.828	503.281	0.000	0.312
	LDL cholesterol	1758.946	1	1758.946	479.435	0.000	0.301
	Total cholesterol	1763.236	1	1763.236	439.055	0.000	0.283
	Alcohol abuse	2303.593	1	2303.593	716.979	0.000	0.392
	Body mass index (BMI)	975.218	1	975.218	180.121	0.000	0.139
	Diet and nutrition	1788.842	1	1788.842	644.169	0.000	0.367
	Drug or substance abuse	2352.308	1	2352.308	846.394	0.000	0.432
	Family history of cancer	1514.191	1	1514.191	353.417	0.000	0.241
	Physical inactivity	1690.900	1	1690.900	522.688	0.000	0.320
	Smoking, tobacco use	2287.430	1	2287.430	803.978	0.000	0.420

	Sun protection	1269.566	1	1269.566	303.638	0.000	0.214
	Insurance coverage	1322.748	1	1322.748	165.165	0.000	0.129
	Hypertension screening	2040.424	1	2040.424	500.233	0.000	0.310
	High school diploma as a health indicator	774.297	1	774.297	100.542	0.000	0.083
	Engaged people (people's attention or efforts are occupied)	877.425	1	877.425	148.816	0.000	0.118
	Major depression	2079.748	1	2079.748	722.685	0.000	0.394
	Having a sense of purpose in one's life	1442.819	1	1442.819	317.041	0.000	0.222
	Race and ethnicity	611.159	1	611.159	94.082	0.000	0.078
	Unemployed individual	1094.338	1	1094.338	184.069	0.000	0.142
Gender	Immunization/Vaccination	9.885	3	3.295	0.681	0.563	0.002
	Personal care needs	15.599	3	5.200	1.147	0.329	0.003
	Cancer screening detection	7.809	3	2.603	0.568	0.636	0.002
	HIV testing	38.621	3	12.874	2.204	0.086	0.006
	Self-rated health status	8.151	3	2.717	0.597	0.617	0.002
	Air quality index > 100	1.662	3	0.554	0.144	0.934	0.000
	Dentist supply	26.372	3	8.791	2.093	0.100	0.006
	Health literacy rate	21.168	3	7.056	1.629	0.181	0.004
	Blood sugar level	13.898	3	4.633	1.601	0.188	0.004
	Blood triglycerides	13.677	3	4.559	1.296	0.274	0.003
	HDL cholesterol	13.467	3	4.489	1.227	0.298	0.003
	LDL cholesterol	26.371	3	8.790	2.396	0.067	0.006
	Total cholesterol	18.733	3	6.244	1.555	0.199	0.004
	Alcohol abuse	41.483	3	13.828	4.304	**0.005**	0.011
	Body mass index (BMI)	66.120	3	22.040	4.071	**0.007**	0.011
	Diet and nutrition	33.095	3	11.032	3.973	**0.008**	0.011
	Drug or substance abuse	29.986	3	9.995	3.597	**0.013**	0.010
	Family history of cancer	17.411	3	5.804	1.355	0.255	0.004
	Physical inactivity	25.582	3	8.527	2.636	**0.048**	0.007
	Smoking, tobacco use	31.365	3	10.455	3.675	**0.012**	0.010
	Sun protection	33.137	3	11.046	2.642	**0.048**	0.007
	Insurance coverage	14.262	3	4.754	0.594	0.619	0.002
	Hypertension screening	11.330	3	3.777	0.926	0.428	0.002
	High school diploma as a health indicator	90.436	3	30.145	3.914	**0.009**	0.010
	Engaged people (people's attention or efforts are occupied)	42.148	3	14.049	2.383	0.068	0.006
	Major depression	17.954	3	5.985	2.080	0.101	0.006
	Having a sense of purpose in one's life	27.525	3	9.175	2.016	0.110	0.005
	Race and ethnicity	34.043	3	11.348	1.747	0.156	0.005
	Unemployed individual	18.794	3	6.265	1.054	0.368	0.003
AgeGroup	Immunization/Vaccination	41.702	4	10.425	2.156	0.072	0.008
	Personal care needs	28.569	4	7.142	1.575	0.179	0.006
	Cancer screening detection	38.528	4	9.632	2.103	0.078	0.008
	HIV testing	12.719	4	3.180	0.544	0.703	0.002
	Self-rated health status	69.331	4	17.333	3.810	**0.004**	0.014
	Air quality index >100	84.799	4	21.200	5.507	**0.000**	0.019
	Dentist supply	59.901	4	14.975	3.565	**0.007**	0.013
	Health literacy rate	17.784	4	4.446	1.026	0.392	0.004
	Blood sugar level	33.741	4	8.435	2.914	**0.021**	0.010
	Blood triglycerides	31.578	4	7.894	2.245	0.062	0.008
	HDL cholesterol	25.408	4	6.352	1.737	0.140	0.006
	LDL cholesterol	39.080	4	9.770	2.663	**0.031**	0.009
	Total cholesterol	16.906	4	4.227	1.052	0.379	0.004
	Alcohol abuse	37.421	4	9.355	2.912	**0.021**	0.010
	Body mass index (BMI)	115.572	4	28.893	5.336	**0.000**	0.019
	Diet and nutrition	74.388	4	18.597	6.697	**0.000**	0.024
	Drug or substance abuse	46.572	4	11.643	4.189	**0.002**	0.015

	Family history of cancer	71.995	4	17.999	4.201	**0.002**	0.015
	Physical inactivity	53.088	4	13.272	4.103	**0.003**	0.015
	Smoking, tobacco use	27.811	4	6.953	2.444	**0.045**	0.009
	Sun protection	141.129	4	35.282	8.438	**0.000**	0.029
	Insurance coverage	24.454	4	6.114	0.763	0.549	0.003
	Hypertension screening	69.174	4	17.294	4.240	**0.002**	0.015
	High school diploma as a health indicator	60.613	4	15.153	1.968	0.097	0.007
	Engaged people (people's attention or efforts are occupied)	170.103	4	42.526	7.213	**0.000**	0.025
	Major depression	13.599	4	3.400	1.181	0.317	0.004
	Having a sense of purpose in one's life	122.867	4	30.717	6.750	**0.000**	0.024
	Race and ethnicity	44.594	4	11.149	1.716	0.144	0.006
	Unemployed individual	28.917	4	7.229	1.216	0.302	0.004
professional group	Immunization/Vaccination	17.890	4	4.472	0.925	0.449	0.003
	Personal care needs	65.599	4	16.400	3.616	**0.006**	0.013
	Cancer screening detection	12.492	4	3.123	0.682	0.605	0.002
	HIV testing	28.228	4	7.057	1.208	0.306	0.004
	Self-rated health status	50.507	4	12.627	2.776	**0.026**	0.010
	Air quality index > 100	20.710	4	5.177	1.345	0.251	0.005
	Dentist supply	20.146	4	5.037	1.199	0.310	0.004
	Health literacy rate	11.035	4	2.759	0.637	0.636	0.002
	Blood sugar level	6.977	4	1.744	0.603	0.661	0.002
	Blood triglycerides	39.750	4	9.937	2.826	**0.024**	0.010
	HDL cholesterol	20.635	4	5.159	1.410	0.228	0.005
	LDL cholesterol	9.177	4	2.294	0.625	0.644	0.002
	Total cholesterol	20.948	4	5.237	1.304	0.267	0.005
	Alcohol abuse	17.174	4	4.293	1.336	0.254	0.005
	Body mass index (BMI)	7.311	4	1.828	0.338	0.853	0.001
	Diet and nutrition	5.415	4	1.354	0.487	0.745	0.002
	Drug or substance abuse	6.074	4	1.518	0.546	0.702	0.002
	Family history of cancer	14.368	4	3.592	0.838	0.501	0.003
	Physical inactivity	3.549	4	0.887	0.274	0.895	0.001
	Smoking, tobacco use	29.353	4	7.338	2.579	**0.036**	0.009
	Sun protection	20.079	4	5.020	1.201	0.309	0.004
	Insurance coverage	105.199	4	26.300	3.284	**0.011**	0.012
	Hypertension screening	16.095	4	4.024	0.986	0.414	0.004
	High school diploma as a health indicator	23.230	4	5.807	0.754	0.555	0.003
	Engaged people (people's attention or efforts are occupied)	39.474	4	9.869	1.674	0.154	0.006
	Major depression	13.697	4	3.424	1.190	0.314	0.004
	Having a sense of purpose in one's life	6.912	4	1.728	0.380	0.823	0.001
	Race and ethnicity	79.821	4	19.955	3.072	**0.016**	0.011
	Unemployed individual	70.891	4	17.723	2.981	**0.018**	0.011
educational qualification	Immunization/Vaccination	17.370	4	4.343	0.898	0.464	0.003
	Personal care needs	20.219	4	5.055	1.115	0.348	0.004
	Cancer screening detection	62.518	4	15.630	3.413	**0.009**	0.012
	HIV testing	83.206	4	20.802	3.562	**0.007**	0.013
	Self-rated health status	37.891	4	9.473	2.083	0.081	0.007
	Air quality index > 100	7.415	4	1.854	0.482	0.749	0.002
	Dentist supply	13.108	4	3.277	0.780	0.538	0.003
	Health literacy rate	23.935	4	5.984	1.381	0.238	0.005
	Blood sugar level	4.274	4	1.069	0.369	0.831	0.001
	Blood triglycerides	6.158	4	1.539	0.438	0.781	0.002
	HDL cholesterol	8.029	4	2.007	0.549	0.700	0.002
	LDL cholesterol	5.229	4	1.307	0.356	0.840	0.001

	Total cholesterol	27.122	4	6.780	1.688	0.150	0.006
	Alcohol abuse	32.277	4	8.069	2.511	**0.040**	0.009
	Body mass index (BMI)	12.762	4	3.190	0.589	0.670	0.002
	Diet and nutrition	24.501	4	6.125	2.206	0.066	0.008
	Drug or substance abuse	14.926	4	3.732	1.343	0.252	0.005
	Family history of cancer	42.200	4	10.550	2.462	**0.044**	0.009
	Physical inactivity	36.633	4	9.158	2.831	**0.024**	0.010
	Smoking, tobacco use	34.605	4	8.651	3.041	**0.017**	0.011
	Sun protection	28.897	4	7.224	1.728	0.141	0.006
	Insurance coverage	37.723	4	9.431	1.178	0.319	0.004
	Hypertension screening	8.617	4	2.154	0.528	0.715	0.002
	High school diploma as a health indicator	21.226	4	5.306	0.689	0.600	0.002
	Engaged people (people's attention or efforts are occupied)	65.306	4	16.327	2.769	**0.026**	0.010
	Major depression	1.003	4	0.251	0.087	0.986	0.000
	Having a sense of purpose in one's life	19.625	4	4.906	1.078	0.366	0.004
	Race and ethnicity	200.057	4	50.014	7.699	**0.000**	0.027
	Unemployed individual	116.453	4	29.113	4.897	**0.001**	0.017
Gender * AgeGroup	Immunization/Vaccination	36.283	6	6.047	1.251	0.278	0.007
	Personal care needs	35.684	6	5.947	1.311	0.249	0.007
	Cancer screening detection	9.918	6	1.653	0.361	0.904	0.002
	HIV testing	3.739	6	0.623	0.107	0.996	0.001
	Self-rated health status	34.892	6	5.815	1.278	0.264	0.007
	Air quality index > 100	90.542	6	15.090	3.920	**0.001**	0.021
	Dentist supply	28.582	6	4.764	1.134	0.340	0.006
	Health literacy rate	52.326	6	8.721	2.013	0.061	0.011
	Blood sugar level	7.882	6	1.314	0.454	0.843	0.002
	Blood triglycerides	23.384	6	3.897	1.108	0.355	0.006
	HDL cholesterol	21.904	6	3.651	0.998	0.425	0.005
	LDL cholesterol	44.268	6	7.378	2.011	0.061	0.011
	Total cholesterol	10.700	6	1.783	0.444	0.849	0.002
	Alcohol abuse	23.912	6	3.985	1.240	0.283	0.007
	Body mass index (BMI)	76.039	6	12.673	2.341	**0.030**	0.012
	Diet and nutrition	68.918	6	11.486	4.136	**0.000**	0.022
	Drug or substance abuse	13.460	6	2.243	0.807	0.564	0.004
	Family history of cancer	5.440	6	0.907	0.212	0.973	0.001
	Physical inactivity	67.970	6	11.328	3.502	**0.002**	0.019
	Smoking, tobacco use	37.475	6	6.246	2.195	**0.041**	0.012
	Sun protection	47.422	6	7.904	1.890	0.079	0.010
	Insurance coverage	94.852	6	15.809	1.974	0.067	0.011
	Hypertension screening	13.817	6	2.303	0.565	0.759	0.003
	High school diploma as a health indicator	12.064	6	2.011	0.261	0.955	0.001
	Engaged people (people's attention or efforts are occupied)	43.730	6	7.288	1.236	0.285	0.007
	Major depression	18.757	6	3.126	1.086	0.368	0.006
	Having a sense of purpose in one's life	79.064	6	13.177	2.896	**0.008**	0.015
	Race and ethnicity	105.364	6	17.561	2.703	**0.013**	0.014
	Unemployed individual	33.176	6	5.529	0.930	0.472	0.005
Gender * professional group	Immunization/Vaccination	71.774	8	8.972	1.855	0.063	0.013
	Personal care needs	107.145	8	13.393	2.953	**0.003**	0.021
	Cancer screening detection	38.816	8	4.852	1.059	0.389	0.008
	HIV testing	68.781	8	8.598	1.472	0.163	0.010
	Self-rated health status	27.381	8	3.423	0.752	0.645	0.005
	Air quality index > 100	39.686	8	4.961	1.289	0.245	0.009
	Dentist supply	30.467	8	3.808	0.907	0.510	0.006
	Health literacy rate	46.465	8	5.808	1.341	0.219	0.010
	Blood sugar level	18.421	8	2.303	0.796	0.607	0.006

	Blood triglycerides	42.369	8	5.296	1.506	0.150	0.011
	HDL cholesterol	33.362	8	4.170	1.140	0.333	0.008
	LDL cholesterol	41.732	8	5.216	1.422	0.183	0.010
	Total cholesterol	46.045	8	5.756	1.433	0.178	0.010
	Alcohol abuse	28.971	8	3.621	1.127	0.342	0.008
	Body mass index (BMI)	22.642	8	2.830	0.523	0.840	0.004
	Diet and nutrition	15.850	8	1.981	0.713	0.680	0.005
	Drug or substance abuse	30.113	8	3.764	1.354	0.213	0.010
	Family history of cancer	54.249	8	6.781	1.583	0.125	0.011
	Physical inactivity	27.170	8	3.396	1.050	0.396	0.007
	Smoking, tobacco use	48.913	8	6.114	2.149	**0.029**	0.015
	Sun protection	62.297	8	7.787	1.862	0.062	0.013
	Insurance coverage	211.562	8	26.445	3.302	**0.001**	0.023
	Hypertension screening	27.723	8	3.465	0.850	0.559	0.006
	High school diploma as a health indicator	76.694	8	9.587	1.245	0.269	0.009
	Engaged people (people's attention or efforts are occupied)	38.893	8	4.862	0.825	0.581	0.006
	Major depression	25.686	8	3.211	1.116	0.350	0.008
	Having a sense of purpose in one's life	25.926	8	3.241	0.712	0.681	0.005
	Race and ethnicity	231.996	8	29.000	4.464	**0.000**	0.031
	Unemployed individual	96.383	8	12.048	2.026	**0.040**	0.014
Error	Immunization/Vaccination	5377.316	1112	4.836			
	Personal care needs	5042.651	1112	4.535			
	Cancer screening detection	5092.958	1112	4.580			
	HIV testing	6494.446	1112	5.840			
	Self-rated health status	5058.166	1112	4.549			
	Air quality index > 100	4281.110	1112	3.850			
	Dentist supply	4671.401	1112	4.201			
	Health literacy rate	4816.817	1112	4.332			
	Blood sugar level	3218.575	1112	2.894			
	Blood triglycerides	3910.417	1112	3.517			
	HDL cholesterol	4067.309	1112	3.658			
	LDL cholesterol	4079.691	1112	3.669			
	Total cholesterol	4465.769	1112	4.016			
	Alcohol abuse	3572.760	1112	3.213			
	Body mass index (BMI)	6020.625	1112	5.414			
	Diet and nutrition	3087.996	1112	2.777			
	Drug or substance abuse	3090.484	1112	2.779			
	Family history of cancer	4764.293	1112	4.284			
	Physical inactivity	3597.326	1112	3.235			
	Smoking, tobacco use	3163.795	1112	2.845			
	Sun protection	4649.474	1112	4.181			
	Insurance coverage	8905.638	1112	8.009			
	Hypertension screening	4535.784	1112	4.079			
	High school diploma as a health indicator	8563.796	1112	7.701			
	Engaged people (people's attention or efforts are occupied)	6556.373	1112	5.896			
	Major depression	3200.122	1112	2.878			
	Having a sense of purpose in one's life	5060.582	1112	4.551			
	Race and ethnicity	7223.575	1112	6.496			
	Unemployed individual	6611.119	1112	5.945			
Total	Immunization/Vaccination	71110.980	1144				
	Personal care needs	60027.050	1144				
	Cancer screening detection	65512.210	1144				
	HIV testing	57301.080	1144				
	Self-rated health status	56088.540	1144				

	Air quality index > 100	56741.850	1144			
	Dentist supply	52268.950	1144			
	Health literacy rate	61725.290	1144			
	Blood sugar level	72557.360	1144			
	Blood triglycerides	65523.797	1144			
	HDL cholesterol	65775.774	1144			
	LDL cholesterol	67399.172	1144			
	Total cholesterol	66352.282	1144			
	Alcohol abuse	78577.780	1144			
	Body mass index (BMI)	55214.850	1144			
	Diet and nutrition	76249.370	1144			
	Drug or substance abuse	86708.090	1144			
	Family history of cancer	57063.540	1144			
	Physical inactivity	72499.780	1144			
	Smoking, tobacco use	86469.860	1144			
	Sun protection	50149.450	1144			
	Insurance coverage	59228.180	1144			
	Hypertension screening	68373.920	1144			
	High school diploma as a health indicator	42209.240	1144			
	Engaged people (people's attention or efforts are occupied)	47015.280	1144			
	Major depression	76446.370	1144			
	Having a sense of purpose in one's life	66823.140	1144			
	Race and ethnicity	36256.540	1144			
	Unemployed individual	47032.010	1144			
Corrected Total	Immunization/Vaccination	5549.330	1143			
	Personal care needs	5286.244	1143			
	Cancer screening detection	5274.813	1143			
	HIV testing	6915.153	1143			
	Self-rated health status	5354.258	1143			
	Air quality index > 100	4519.753	1143			
	Dentist supply	5040.060	1143			
	Health literacy rate	5036.112	1143			
	Blood sugar level	3413.436	1143			
	Blood triglycerides	4073.421	1143			
	HDL cholesterol	4259.551	1143			
	LDL cholesterol	4325.687	1143			
	Total cholesterol	4672.224	1143			
	Alcohol abuse	3866.220	1143			
	Body mass index (BMI)	6702.586	1143			
	Diet and nutrition	3322.684	1143			
	Drug or substance abuse	3360.742	1143			
	Family history of cancer	5169.287	1143			
	Physical inactivity	3899.835	1143			
	Smoking, tobacco use	3567.474	1143			
	Sun protection	5340.618	1143			
	Insurance coverage	9416.658	1143			
	Hypertension screening	4846.276	1143			
	High school diploma as a health indicator	8908.495	1143			
	Engaged people (people's attention or efforts are occupied)	7360.024	1143			
	Major depression	3366.307	1143			
	Having a sense of purpose in one's life	5473.331	1143			
	Race and ethnicity	8152.372	1143			
	Unemployed individual	7104.281	1143			

a. R Squared = .031 (Adjusted R Squared = .004)
b. R Squared = .046 (Adjusted R Squared = .019)
c. R Squared = .034 (Adjusted R Squared = .008)

d. R Squared = .061 (Adjusted R Squared = .035)
e. R Squared = .055 (Adjusted R Squared = .029)
f. R Squared = .053 (Adjusted R Squared = .026)
g. R Squared = .073 (Adjusted R Squared = .047)
h. R Squared = .044 (Adjusted R Squared = .017)
i. R Squared = .057 (Adjusted R Squared = .031)
j. R Squared = .040 (Adjusted R Squared = .013)
k. R Squared = .045 (Adjusted R Squared = .019)
l. R Squared = .057 (Adjusted R Squared = .031)
m. R Squared = .044 (Adjusted R Squared = .018)
n. R Squared = .076 (Adjusted R Squared = .050)
o. R Squared = .102 (Adjusted R Squared = .077)
p. R Squared = .071 (Adjusted R Squared = .045)
q. R Squared = .080 (Adjusted R Squared = .055)
r. R Squared = .078 (Adjusted R Squared = .053)
s. R Squared = .078 (Adjusted R Squared = .052)
t. R Squared = .113 (Adjusted R Squared = .088)
u. R Squared = .129 (Adjusted R Squared = .105)
v. R Squared = .054 (Adjusted R Squared = .028)
w. R Squared = .064 (Adjusted R Squared = .038)
x. R Squared = .039 (Adjusted R Squared = .012)
y. R Squared = .109 (Adjusted R Squared = .084)
z. R Squared = .049 (Adjusted R Squared = .023)
aa. R Squared = .075 (Adjusted R Squared = .050)
ab. R Squared = .114 (Adjusted R Squared = .089)
ac. R Squared = .069 (Adjusted R Squared = .043)
